# A broadly cross-reactive monoclonal antibody against hepatitis E virus capsid antigen

**DOI:** 10.1007/s00253-021-11342-7

**Published:** 2021-06-15

**Authors:** Barbara Kubickova, Jörg A. Schenk, Franziska Ramm, Kornelija Markuškienė, Jochen Reetz, Paul Dremsek, Paulius Lukas Tamosiunas, Laima Cepulyte, Hoai Anh Trinh, Johannes Scholz, Henry Memczak, Marc Hovestädt, René Ryll, Rasa Petraityte-Burneikiene, Victor M. Corman, Anika Andersson, Dietmar Becher, Martin H. Groschup, Stefan Kubick, Frank Sellrie, Reimar Johne, Rainer G. Ulrich

**Affiliations:** 1 Institute of Novel and Emerging Infectious Diseases, Friedrich-Loeffler-Institut, Federal Research Institute for Animal Health, 17493 Greifswald-Insel Riems, Germany; 2 Present Address: RECETOX, Faculty of Science, Masaryk University, 62500 Brno, Czech Republic; 3 Hybrotec GmbH, 14476 Potsdam, Germany; 4 UP Transfer GmbH an der Universität Potsdam, 14469 Potsdam, Germany; 5 Branch Bioanalytics and Bioprocesses (IZI-BB), Fraunhofer Institute for Cell Therapy and Immunology (IZI), 14476 Potsdam, Germany; 6 Institute of Chemistry and Biochemistry, Freie Universität Berlin, 14195 Berlin, Germany; 7 Institute of Biotechnology, Life Sciences Centre, Vilnius University, 02241 Vilnius, Lithuania; 8 German Federal Institute for Risk Assessment, 10589 Berlin, Germany; 9 Present Address: Zentrum für Pathobiochemie und Genetik, Medizinische Universität Wien, 1090 Wien, Austria; 10 qpa bioanalytics GmbH, 10585 Berlin, Germany; 11 Present Address: Surflay Nanotec, 12489 Berlin, Germany; 12 Institute of Virology, Charité – Universitätsmedizin Berlin, 10117 Berlin, Germany; 13 Site Berlin, German Center for Infection Research (DZIF), 10117 Berlin, Germany; 14 Partner site Hamburg-Lübeck-Borstel-Riems, German Center for Infection Research (DZIF), 17493 Greifswald-Insel Riems, Germany; 15 Micromun GmbH, 17489 Greifswald, Germany; 16 Faculty of Health Sciences, Joint Faculty of the Brandenburg University of Technology Cottbus–Senftenberg, the Brandenburg Medical School Theodor Fontane and the University of Potsdam, Potsdam, Germany

**Keywords:** Hepatitis E virus, HEV-1, HEV-2, HEV-3, HEV-4, HEV-7, ratHEV, batHEV, cvHEV, Monoclonal antibody, Cross-reactivity, Cell-free synthesis

## Abstract

**Abstract:**

To generate a hepatitis E virus (HEV) genotype 3 (HEV-3)–specific monoclonal antibody (mAb), the *Escherichia coli*–expressed carboxy-terminal part of its capsid protein was used to immunise BALB/c mice. The immunisation resulted in the induction of HEV-specific antibodies of high titre. The mAb G117-AA4 of IgG1 isotype was obtained showing a strong reactivity with the homologous *E. coli*, but also yeast-expressed capsid protein of HEV-3. The mAb strongly cross-reacted with ratHEV capsid protein derivatives produced in both expression systems and weaker with an *E. coli*–expressed batHEV capsid protein fragment. In addition, the mAb reacted with capsid protein derivatives of genotypes HEV-2 and HEV-4 and common vole hepatitis E virus (cvHEV), produced by the cell-free synthesis in Chinese hamster ovary (CHO) and *Spodoptera frugiperda* (*Sf*21) cell lysates. Western blot and line blot reactivity of the mAb with capsid protein derivatives of HEV-1 to HEV-4, cvHEV, ratHEV and batHEV suggested a linear epitope. Use of truncated derivatives of ratHEV capsid protein in ELISA, Western blot, and a Pepscan analysis allowed to map the epitope within a partially surface-exposed region with the amino acid sequence LYTSV. The mAb was also shown to bind to human patient–derived HEV-3 from infected cell culture and to hare HEV-3 and camel HEV-7 capsid proteins from transfected cells by immunofluorescence assay. The novel mAb may serve as a useful tool for further investigations on the pathogenesis of HEV infections and might be used for diagnostic purposes.

**Key points:**

*• The antibody showed cross-reactivity with capsid proteins of different hepeviruses.*

*• The linear epitope of the antibody was mapped in a partially surface-exposed region.*

*• The antibody detected native HEV-3 antigen in infected mammalian cells.*

**Supplementary Information:**

The online version contains supplementary material available at 10.1007/s00253-021-11342-7.

## Introduction

Hepatitis E virus (HEV) was discovered as the causative agent of acute non-A-non-B hepatitis at the beginning of the 1980s (Balayan et al. 1983). For many years, four different genotypes have been known to be involved in disease cases in humans. The genotypes 1 (HEV-1) and 2 (HEV-2) were found in endemic regions with lower sanitary conditions, whereas the genotype 3 (HEV-3) was found to be distributed worldwide also affecting industrialised countries (Scobie and Dalton 2013). Genotype 4 (HEV-4) seems to have a restricted geographic distribution in Asia. Whereas HEV-1 and HEV-2 are thought to be mainly transmitted faecal-orally by contaminated water resources, the other two genotypes are zoonotic with wild boar, domesticated pig and deer representing the reservoirs (Johne et al. 2014a). Recently, further HEV genotypes, subgenotypes and strains have been identified in rabbit, hare, wild boar, moose, camels and other animals (Lin et al. 2015; Pavio et al. 2015; Sato et al. 2011; Schlosser et al. 2014; Takahashi et al. 2014; Woo et al. 2014; Corman et al. 2019).

In addition to human pathogenic HEV, related viruses have been identified in fowl, rats, bats, carnivores and even in fish (Batts et al. 2011; Bodewes et al. 2013; Drexler et al. 2012; Johne et al. 2010a, b; Krog et al. 2013; Raj et al. 2012). Due to the results of experimental transmission studies, these novel viruses were initially thought to have no zoonotic potential (Johne et al. 2014a), although ratHEV-reactive antibodies have been demonstrated in forestry workers from Germany and in fever patients in China (Dremsek et al. 2012; Shimizu et al. 2016). ratHEV has been detected in rats of different species, but also in non-rodent species, i.e. shrews (Guan et al. 2013; Johne et al. 2010a; Li et al. 2013). After its initial description in Germany, ratHEV has been detected in many regions of the world suggesting a worldwide distribution (Johne et al. 2010b, 2012; Mulyanto et al. 2014; Purcell and Emerson 2008; Ryll et al. 2017; Wolf et al. 2013). Recently, several ratHEV-caused human disease cases have been reported (Andonov et al. 2019; Sridhar et al. 2018, 2021). In addition to the rat-associated HEV of genotype C1, additional hepeviruses have been discovered in rodents (Wang et al. 2017), including a virus associated to the common vole *Microtus arvalis* (Ryll et al. 2019).

According to the International Committee on Taxonomy of Viruses (ICTV), all these viruses belong to the family *Hepeviridae* (Meng et al. 2012). The family is subdivided into two genera, *Orthohepevirus* and *Piscihepevirus* (Smith et al. 2014; Walker et al. 2019), with the latter comprising only the fish-associated viruses (Batts et al. 2011). The genus *Orthohepevirus* comprises the human pathogenic and related genotypes within the species *Orthohepevirus A*, the avian strains as *Orthohepevirus B*, the rodent and carnivore strains as *Orthohepevirus C* and the bat strains as *Orthohepevirus D*.

**Fig. 1:**
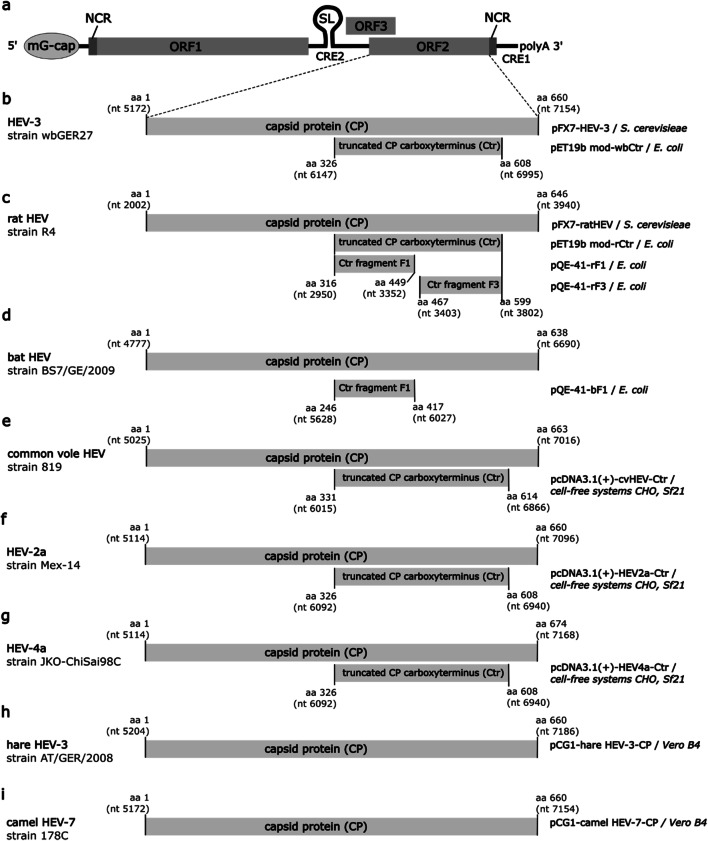
Genome organization of hepatitis E virus (HEV) (**a**) and schematic structure of the capsid proteins of HEV-3 (**b**), ratHEV (**c**), batHEV (**d**), common vole HEV (**e**), HEV-2a (**f**), HEV-4a (**g**), hare HEV-3 (**h**) and camel HEV-7 (**i**). Corresponding expression vectors and expression systems are indicated on the right. GenBank accession numbers: HEV-3 wbGER27, FJ705359.1; rat HEV strain R4, GQ504009.1; batHEV strain BS7/GE/2009, JQ001749.1; cvHEV strain 819, MK192409.1; HEV-2a strain Mex-14, KX578717.1; HEV-4a strain JKO-ChiSai98C, AB197673.1; hare HEV-3 strain AT/GER/2008, MK050463.1; camel HEV-7 strain 178C, KJ496143.1. mG-cap, 7-methylguanylate cap. NCR: non-coding region. ORF: open reading frame. SL: stem-loop structure. CRE: cis-regulatory element. polyA: polyadenylate tail. aa: amino acid. nt: nucleotide. HEV-2: HEV genotype 2. HEV-3: HEV genotype 3. HEV-4: HEV genotype 4. HEV-7: HEV genotype 7. CP: capsid protein, Ctr: carboxy-terminal truncated capsid protein, *CHO*: Chinese hamster ovary, *Sf*21: *Spodoptera frugiperda* 21. Genome organization of HEV in panel a according to Cao and Meng (2012)

Hepeviruses are thought to be non-enveloped; however, membrane structures have been found to be associated with virions in a subset of cell culture–derived HEV and HEV from the serum of patients (Nagashima et al. 2014). The capsid consists of the capsid protein encoded by the open reading frame (ORF) 2. An additional, partially overlapping ORF3 encodes a small phosphoprotein which is associated with the virion and involved in virus release (Cao and Meng 2012; Zafrullah et al. 1997). The ORF1 located at the 5′-end of the genome encodes a polyprotein comprising several nonstructural proteins including regions with similarity to methyltransferases, papain-like proteases, helicases and RNA-dependent RNA polymerases (Johne et al. 2014a) (see Fig. 1a).

The capsid protein represents the primary target for antibodies in infected individuals and therefore is the most frequently used antigen for diagnostic tests (Khudyakov and Kamili 2011). The recombinant capsid protein produced by baculovirus expression systems or yeast was found to form virus-like particles (VLPs) (Li et al. 1997, 2005a, b, 2011; Simanavicius et al. 2018). Capsid protein immunisation resulted in the induction of protective immunity in animal models (Li et al. 2004; Tsarev 1997). Furthermore, a VLP-based vaccine was developed which elicited virus-neutralising antibodies in human volunteers (Li et al. 2005a). Structural and epitope mapping studies revealed that major neutralising epitopes are located within the C-terminal part of the capsid protein, forming outward-extending protrusions (P domain, amino acid residues 456–606 in the case of HEV-3) (Xing et al. 1999; Yamashita et al. 2009; Zhao et al. 2015).

In the present study, we generated a novel mAb against *E. coli*–expressed HEV-3 capsid protein, which demonstrated a broad cross-reactivity to the capsid protein of other HEV species and strains and reacted with the native viral antigen in different test formats.

## Materials and methods

### Construction of HEV capsid protein expression plasmids

The construction of pET19b-mod-derived *E. coli* expression plasmids encoding the carboxy-terminal part of the capsid proteins (Ctr) of HEV-3, strain wbGER27 (Schielke et al. 2009) (GenBank accession number FJ705359.1), and of ratHEV strain R4 (Johne et al. 2010b) (GenBank accession number: GQ504009.1) has been described previously by Dremsek et al. 2012 (see Fig. 1b, c). Yeast-expressed ratHEV and HEV-3 capsid proteins were obtained as described previously (Simanavicius et al. 2018).

The ratHEV Ctr encoding region was further fragmented into two pieces F1 and F3 (Fig. 1c) and subcloned into pQE-41 expression vector (Qiagen, Hilden, Germany) by in-fusion cloning (In-Fusion HD Cloning Kit, Clontech Laboratories, CA, USA) using the primers denoted in Table 1. PCR amplification of batHEV Ctr fragment F1 (Fig. 1d) was done using the strain BatHEV/BS7/GE/2009 (Drexler et al. 2012; GenBank accession number JQ001749.1) as a template. The PCR product was inserted into *Kpn*I-linearized pQE-41 plasmid. The resulting pQE-41-derived proteins contain N-terminal His-tags and dihydrofolate reductase (DHFR) sequences.

**Table 1:** Sequences of primers used for construction and sequencing of HEV-3, rat HEV and bat HEV capsid protein derivatives. *italic*, restriction site KpnI/XbaI; **bold**, viral sequence

**Target**	**Strain**	**Designation of oligonucleotide**	**Sequence of oligonucleotide**	**Reference**
HEV-3	wbGER27	wbCtr fw	5’-TATA*TCTAGA*ACAATG**ACAGCCCGTCATCGGCTGCGCCGCGGTGCTG**-3’	Dremsek et al. 2012
		wbCtr rev	5’-TATA*TCTAGA*TTA**TGCAAGAGCCGAATGTGGGGCTAAAACAAC**-3’	
rat HEV	R4	rCtr fw	5'-TATA*TCTAGA*ACAATG**ACAGCCCCGCATAAGATCAAGCGGCT**-3'	Dremsek et al. 2012
		rCtr rev	5'-TATA*TCTAGA*TTA**CTGCTCAGTCGGGCTGGGGCCGATA**-3'	
		rHE_fw_Ctr	5'-TC*AGATCT*GCATGCGGTACC**AGCCCCGCATAAGATCAAG**-3'	This study
		rHE_re_Ctr	5'-GCAGGTCGACCCGGGGTACCTTA**CTGCTCAGTCGGGCTGG**-3'	
		rHE_re_F1	5'-GCAGGTCGACCCGGGGTACCTTA**GCGAGCGGGCGCAGGG**-3'	
		rHE_fw_F3	5'-TC*AGATCT*GCATGCGGTACC**AGAGTATGCCCAATCGCAG**-3'	
bat HEV	batHEV/BS7/GE/2009	bF1 fw	5'-TATA*GGTACC***AACTCAGCACCGCCTGACCCGCGGG**-3'	This study
		bF1 rev	5'-TATA*GGTACC***TTAACGCTTCGGGGCAGGCGCAGGTGA**-3'	
pQE	-	pQE_insert_fw	5'-AGGAATTGAAAGTGACACG-3'	Qiagen
pQE	-	pQE_insert_rev	5'-ATTACTGGATCTATCAACAGG-3'	Qiagen

The inserted nucleotide sequences were confirmed by sequencing using the BigDye® Terminator v1.1 Cycle Sequencing Kit (Life Technologies, Carlsbad, CA, USA) and primers pQE-insert fw and pQE-insert rev (Table 1) on a 3130xl Genetic Analyser (Applied Biosystems®, Life Technologies GmbH, Darmstadt, Germany).

The codon-optimized entire capsid protein coding sequence of hare HEV-3 (GenBank accession number MK050463.1) has been inserted into the eukaryotic expression plasmid pCG1 (Corman et al. 2019; Fig. 1h). The entire capsid protein coding sequence of camel-associated HEV-7 (GenBank accession number KJ496143.1; Fig. 1i) was inserted in the same way into this expression plasmid and confirmed by re-sequencing.

### Expression and purification of HEV capsid protein derivatives in *E. coli*

Expression of pET19b-mod-encoded proteins of HEV-3 and ratHEV was performed in *E. coli* BL21(DE3) cells in LB medium with 100 mg/l ampicillin. The pQE-encoded DHFR-ratHEV and -batHEV fusion proteins were expressed in *E. coli* M15[pREP4] in LB medium with 100 mg/l ampicillin and 50 mg/l kanamycin as described by Dremsek et al. (2012). Analysis for proteins of the expected size was performed by sodium dodecyl sulphate polyacrylamide gel electrophoresis (SDS-PAGE) and Coomassie Brilliant Blue staining, as well as Western blot analysis with His•Tag® mAb (Novagen, EMD Chemicals, Merck, Darmstadt, Germany). His-tagged recombinant proteins were purified by immobilised metal affinity chromatography (Ni-NTA sepharose) under denaturing conditions according to the protocol of the manufacturer (Qiagen, Hilden, Germany). After purification, protein concentration was determined by Bradford assay (Roth, Karlsruhe, Germany) with bovine serum albumin (BSA) used as a standard.

### Generation of HEV-specific monoclonal antibodies and rabbit polyclonal serum

The HEV-specific mAb was generated by hybridoma technology (Köhler and Milstein 1975). For this purpose, 10-week-old female Balb/c mice were immunised three times with *E. coli*–expressed HEV-3 Ctr protein. Immunisation started with 100 μg recombinant protein using Freund’s complete adjuvant. Booster immunisations were carried out 5 and 12 weeks after the first immunisation using 50 μg purified protein without adjuvant. Four days after the final booster immunisation, electrofusion of spleen cells with myeloma cells (P3X63Ag8.653, ATCC CRL-1580) in the presence of polyethylene glycol 8000 was performed as described (Schenk et al. 2004). Selected hybrids were cultivated in RPMI 1640 medium (containing 10% foetal calf serum (FCS), 2 mM glutamine and 50 mM β-mercaptoethanol) and subcloned by limiting dilution on mouse peritoneal feeder cells. Culture supernatants of clones and subclones were tested in an indirect enzyme-linked immunosorbent assay (ELISA) for antigen binding on recombinant HEV-3 and ratHEV proteins adsorbed to microtitre plates. The class and subclass of the mAb were determined in another indirect ELISA. In brief, recombinant HEV-3 Ctr protein (5 μg/ml in phosphate-buffered saline (PBS)) was adsorbed to a microtitre plate. After blocking with PBS/5% neonatal calf serum (NCS, Biochrom, Berlin, Germany), the culture supernatant of the mAb was added to the plate. After incubation for 1 h, biotinylated class- and subclass-specific antibodies (Serva, Heidelberg, Germany) were added, followed by streptavidin-horseradish peroxidase (HRP) conjugate. Staining was done by addition of 3,3′,5,5′-tetramethylbenzidine (TMB). Reaction was stopped after 10 min and absorption measured at 450 nm.

To obtain larger amounts of this antibody, hybridoma cells were adapted to serum-free medium and fermentation conditions and the antibodies were purified from culture supernatant via protein A affinity chromatography to avoid contamination with secretory leukocyte protease inhibitor (Schenk et al. 2012).

A polyclonal serum was produced by subcutaneous immunizations of a rabbit with *E. coli*–expressed and affinity chromatography purified HEV-3 Ctr capsid protein derivative (Dremsek et al. 2012). This serum has been successfully used in immune electron microscopy (Berto et al. 2013) and immunofluorescence assays (Johne et al. 2016; Schemmerer et al. 2016).

### ELISA and line assay

Characterisation of the mAb was performed using a commercially available *recom*Line HEV assay with HEV-1 and HEV-3 antigens (Mikrogen, Neuried, Germany) and in-house assays using recombinant HEV-3, ratHEV and batHEV antigens produced in *E. coli*. The commercial assay was performed according to the protocol of the manufacturer, except that HRP-labelled anti-mouse IgG (Blotting Grade Affinity Purified Goat Anti-Mouse IgG (H+L) HRP Conjugate, Bio-Rad, Munich, Germany), diluted 1:2000, was used.

Antibody titrations in the in-house ELISAs were carried out following a modified protocol of Dremsek et al. (2012). Briefly, a microtitre plate (MaxiSorp, Nunc, Thermo Fisher Scientific, Waltham, MA, USA) was coated overnight at 4 °C with 100 μl per well of 1.0 μg/ml purified recombinant antigen in carbonate buffer or plain carbonate buffer. After initial binding of the antigens, free binding sites of the plate were blocked with 200 μl/well of 1.0 % (w/v) BSA in PBS with 0.05 % Tween 20 (0.05% PBS-T) for 1 h at room temperature. The blocking buffer was then replaced by 100 μl primary antibody in binding buffer (0.5 % (w/v) BSA in 0.05% PBS-T) and incubated for 1 h at 37 °C. Each well was subsequently washed three times with 250 μl 0.1% PBS-T and incubated for 1 h at 37 °C with 100 μl of HRP-coupled secondary antibody (Blotting Grade Affinity Purified Goat Anti-Mouse IgG (H+L) HRP Conjugate, Bio-Rad), diluted 1:5000 in binding buffer. Before application of the peroxidase substrate TMB (Bio-Rad) with 100 μl/well, the plate was washed three times with 250 μl 0.1% PBS-T per well. The chromogenic reaction of TMB with HRP was carried out for 10 min at room temperature in the dark, before being stopped by addition of 100 μl 1 M sulphuric acid per well. For quantification of the colour reaction, the absorbance at 450 nm was determined on a microtitre plate reader (Infinite M200 Pro, Tecan, Salzburg, Austria). Data were evaluated in Excel 2007 (Microsoft Office), and non-linear regression with least-squares fit to obtain EC_50_-values as a parameter of antigen-binding was performed using GraphPad Prism (v. 5.00 for Windows, GraphPad Software, San Diego California USA, www.graphpad.com).

### SDS-PAGE and Western blot analysis

For Western blot analysis, purified recombinant proteins (1 μg protein/lane) of HEV-3, ratHEV and batHEV or persistently HEV-3-infected cells were separated by discontinuous denaturing SDS-PAGE (Green and Sambrook 2012).

After transfer of the proteins onto a polyvinylidenfluoride (PVDF) membrane (Immobillon P, EMD Millipore, Billerica, MA, USA) by semidry Western blotting for 90 min at 20 V and 0.18 mA per cm^2^ of gel size, free binding sites were blocked with 5 % (w/v) skim milk in 0.1% PBS-T at 4 °C overnight. Incubation with primary antibody (mAb G117-AA4: 0.25 μg/ml; mouse anti-human-β-actin antibody (8H10D10, Cell Signaling, Beverly, MA, USA): 1:10,000) was carried out in blocking buffer for 1 h, gently agitating at room temperature, followed by three washing steps with 10 ml PBS-T for 10 min. HRP-coupled secondary antibody (Blotting Grade Affinity Purified Goat Anti-Mouse IgG (H+L) HRP Conjugate, Bio-Rad) was diluted 1:2000 in 0.1% PBS-T and incubated gently agitated at room temperature for 1 h. Subsequently, the membrane was washed twice with 10 ml 0.1% PBS-T for 10 min and twice with 10 ml PBS for 10 min, before the peroxidase substrate was applied to the membrane. Chemiluminescence using Pierce ECL Western Blotting Substrate (Thermo Fisher Scientific, Waltham, MA, USA) was detected in a VersaDoc 400MP (Bio-Rad) with an exposure time of 120 s.

### Cell-free synthesis of ORF2-encoded proteins of cvHEV, HEV-2 and HEV-4

Cell-free synthesis reactions using translationally active lysates derived from Chinese hamster ovary (CHO) cells and *Spodoptera frugiperda* 21 (*Sf*21) cells were performed as previously described (Brödel et al. 2013, 2014; Stech et al. 2012; Thoring et al. 2016). Plasmids encoding the C-terminal region of the cvHEV strain 11_819 (GenBank accession number MK192409) capsid protein (Fig. 1e) were designed according to Brödel et al. (2013). In this study, two plasmids were used: one harbouring the coding gene sequence alone and a second plasmid encoding an additional melittin (Mel) signal sequence at the N-terminus of the capsid protein. The Mel-signal sequence was used to facilitate the translocation of the cvHEV protein into the microsomal vesicles present in the eukaryotic lysate. Hence, possible glycosylation patterns of the protein could be analysed. Genes were obtained by de novo gene synthesis (BioCat GmbH). Sequences were cloned into the pcDNA3.1(+) vector backbone by BioCat GmbH, and the plasmids were directly used as a template in cell-free protein synthesis. In a further evaluation, gene fragments of HEV-2a (GenBank accession number KX578717.1, amino acid residues 326-608) and HEV-4a (GenBank accession number AB197673.1, amino acid residues 326-608) were tested in eukaryotic cell-free systems. Therefore, gene blocks generated by Integrated DNA Technologies IDT (Coralville, IA, USA) harbouring the Mel signal peptide were used for cell-free protein synthesis (see Fig. 1f and 1g).

Protein synthesis was conducted in coupled transcription/translation reactions in a final volume of 25 μl using either CHO or *Sf*21 lysate. Batch-based reactions were incubated in a thermomixer (Eppendorf, Hamburg, Germany) for 3 h at 30 °C (CHO) or 27 °C (*Sf*21) and 500 rpm. Cell-free synthesis reactions were composed of 40% (v/v) translationally active lysate supplemented with HEPES-KOH (30 mM, pH 7.6, Carl Roth GmbH, Karlsruhe, Germany), sodium acetate (100 mM, Merck, Darmstadt, Germany), Mg(OAc)_2_ (3.9 mM, Merck), KOAc (150 mM, Merck), amino acids (complete 100 μM, Merck), spermidine (0.25 mM; Roche) and energy regenerating components including creatine phosphokinase (0.1 mg/ml, Roche), creatine phosphate (20 mM, Roche), ATP (1.75 mM, Roche) and GTP (0.3 mM, Roche), 1 U/μl T7 RNA polymerase, 0.3 mM of UTP (Roche), CTP (Roche) and 0.1 mM of the cap analogue m7G(ppp)G (Prof. Edward Darzynkiewicz, Warsaw University, Poland). PolyG primer (12 μM, IBA) was additionally supplemented. For further analyses including autoradiography, cell-free protein synthesis reactions were supplemented with radioactive ^14^C-leucine (50 μM, specific radioactivity 66.67 dpm/pmol, Perkin Elmer).

After 3 h of incubation, the crude translation mixture (TM) was centrifuged (16,000 × g, 10 min, 4 °C) resulting in the supernatant (SN) containing the soluble proteins that were synthesised without Mel-signal sequence and the pelleted microsomes containing the translocated proteins harbouring a Mel-signal peptide. The pellet was resuspended in PBS resulting in the microsomal fraction (MF).

### Western blot analysis of proteins produced by cell-free synthesis

For qualitative analysis, the SN fraction of proteins without Mel-signal peptide and the MF of proteins with Mel-signal peptide were analysed. Protein samples were analysed in native and denatured form. To study the binding of the G117-AA4 antibody to native proteins, 10 μl of SN or MF were mixed with 10 μl of 2 × LDS sample buffer (NUPAGE LDS sample buffer) and directly loaded onto the gel. Further, 10 μl aliquots of the fraction of interest were precipitated in cold acetone (Carl Roth GmbH) and handled as described previously (Thoring et al. 2019). SDS-PAGE using precast (NuPAGE, 10% Bis-Tris, Life technologies) and self-cast (10% Tris-Glycine, Life technologies) were performed. SeeBlue Plus2 Pre-Stained marker was used as a weight measurement. Afterwards, Western blotting was performed using the iBlot Gel transfer device. Proteins were blotted on a PVDF membrane with 20 V for 10 min. The membranes were washed with TBS/T for 5 min, repeated twice and incubated overnight at 4°C with blocking buffer (2% BSA in TBS/T). On the next day, three washing steps with TBS/T were implemented before incubating the membrane with antibody G117-AA4 (1:1000, 2% BSA in TBS/T) or the polyclonal rabbit serum (see above) for 3 h at room temperature on an orbital shaker at 60 rpm. The washing procedure in TBS/T was repeated, followed by addition of the secondary HRP-linked anti-mouse IgG antibody (1:2000, 2% BSA in TBS/T, Cell Signalling Technologies, Beverly, MA, USA) for the monoclonal antibody and a HRP-linked anti-rabbit antibody (1:1000, 2% BSA in TBS/T, Cell Signalling Technologies) for the polyclonal rabbit serum, and incubation of the membrane for 1 h. After a final washing procedure, the membranes were incubated with a chromogenic 4-chloro-1-naphtol/MeOH/PBS/H_2_O_2_ solution to visualize the immune reaction for the initial Western blot analysis of cvHEV-derived capsid protein derivatives. The immune reaction of cvHEV, HEV-2a and HEV-4a capsid proteins was visualized with chemiluminescent WesternBright ECL HRP substrate (Advanstra, San Jose, CA, USA) and detected with the Azure c600 Gel Imaging System (Azure Biosystems, Dublin, CA, USA). At last, the membranes were dried at 70 °C (Unigeldryer 3545D, Uniequip, Planegg, Germany), placed on a phosphor screen (GE Healthcare, Freiburg, Germany) and radioactively labelled proteins were visualised using a Typhoon Trio + variable mode imager (GE Healthcare).

### Cell culture propagation of HEV-3

The HEV-3 strain 47832c was originally isolated from the serum of a chronically infected transplant patient by inoculation onto the human lung carcinoma cell line A549 (ATCC-CCL-185^TM^). By this, a cell line persistently infected with the HEV strain was established (Johne et al. 2014b). These cells were seeded and grown for 14 days as described (Schemmerer et al. 2016), and subsequently analysed by immunofluorescence and Western blot assays. Non-infected A549 cells served as control.

### Immunofluorescence analysis

Persistently HEV-3-infected A549 cells and non-infected A549 cells were fixed using acetone/methanol (1:1) after growth for 14 days at 34.5 °C in 24-well tissue culture plates. After washing with PBS, cells were treated with 1 ml PBS supplemented with 1% FCS for 10 min at 37 °C. After that, 100 μl PBS supplemented with 1% FCS and containing the antibody G117-AA4 in a dilution of 1:100 (corresponding to 0.66 μg) were added. After incubation for 1 h at 37 °C, cells were washed three times with PBS and 100 μl of anti-mouse IgG-fluorescein isothiocyanate conjugate (Sigma, Deisenhofen, Germany), diluted 1:200 in PBS with 1% FCS was added. After further incubation for 1 h at 37 °C, cells were washed twice with PBS and once with double-distilled water and mounted using Roti®-Mount Fluor Care DAPI (Carl Roth GmbH, Karlsruhe, Germany). Fluorescence was observed under an Axio Observer Z1 microscope (Carl Zeiss Microscopy GmbH, Jena, Germany). By this, HEV antigen shows green fluorescence, whereas the cell nuclei are stained in blue.

For analysis of the capsid proteins of hare HEV-3 and camel HEV-7, Vero B4 cells were transfected by the corresponding pCG1-derived expression plasmids. Immunofluorescence analyses with mAb G117-AA4 was performed essentially as described previously (Corman et al. 2019), but stained with Alexa 488-labeled anti-mouse IgG. The mAb was used in a dilution of 1:100.

### Pepscan analysis

Epitope mapping with the protein sequence of the F1-fragment of ratHEV was performed using surface-based peptide synthesis, following an adapted F_moc_-based peptide synthesis protocol (Frank 1992) to generate the microarray with an automated spotting robot (Slide Spotting Robot, Intavis Bioanalytical Instruments AG, Cologne, Germany). The synthesis was performed on amino-modified glass slides, followed by primary antibody G117-AA4 incubation at 50 nM in PBS-T buffer for several hours or overnight. Subsequently, after washing, secondary Alexa Fluor 647–labelled goat anti-mouse antibody (115-605-071, Jackson ImmunoResearch Laboratories, Inc., West Grove, PA, USA) was incubated for 2 h at 750 ng/ml. After washing and drying, the fluorescence signal was detected using a GenePix Axon 4000A (Molecular Devices, Sunnyvale, CA, USA) fluorescence scanner.

### Amino acid sequence comparison

Amino acid sequences were obtained by in silico translation of ORF2 nucleotide sequences of HEV-3, strain wbGer27, ratHEV strains R4 and R63, batHEV strain BS7 GE 2009, cvHEV strain 11_819/CZE/2010, HEV-1, HEV-2, HEV-4, hare HEV-3, camel HEV-7 and additional reference sequences of *Orthohepevirus A* and *Orthohepevirus C* genotypes (Smith et al. 2014, 2016, 2020) (GenBank accession numbers are given in Table 2). The resulting amino acid sequences were aligned using ClustalW algorithm in BioEdit software (version 7.0.5, www.mbio.ncsu.edu/BioEdit/bioedit.htm).

**Table 2:**
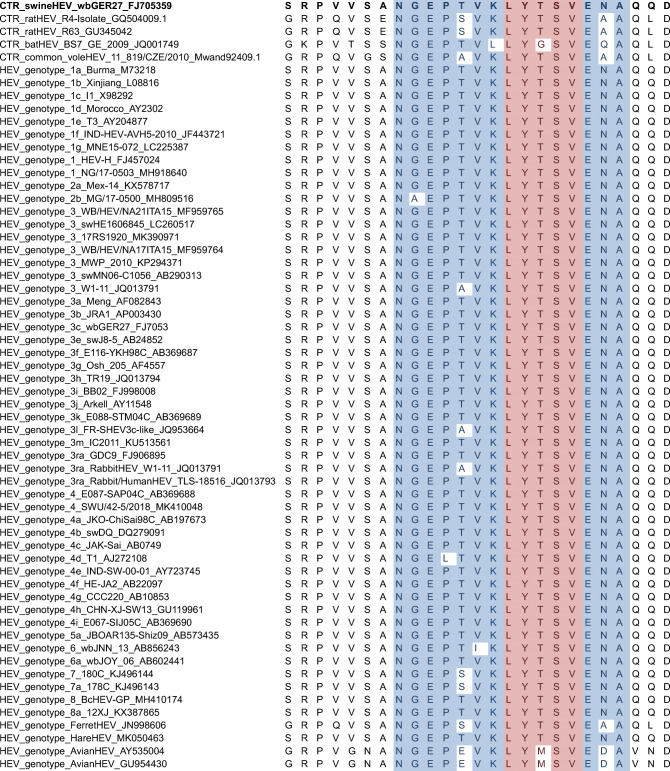
Multiple sequence alignment of the assumed G117-AA4 epitope region of different HEV genotypes. The amino acid sequence NGEPSVKLYTSVEAA (shaded, blue) showed very high affinity to mAb G117-AA4 in Pepscan analysis with the peptide LYTSV (shaded, red) being the major epitope region. CTR_swineHEV_wbGER27_ FJ705359 (bold) was used for generation of mAb G117-AA4

## Results

### Generation and initial characterisation of a HEV-3-specific monoclonal antibody

Immunisation of two mice with recombinant HEV-3 Ctr protein expressed in *E. coli* led to an ELISA titre ranging from 1:10,000 to 1:20,000. The electrofusion of spleen cells with myeloma cell line P3X63Ag8.653 yielded in more than 1000 hybridoma clones of which 12 clones reacted in the ELISA with *E. coli*–expressed HEV-3 Ctr protein as antigen. Clone G117-AA4 was eventually established as a monoclonal cell line and chosen for further investigations. The isotype of the antibody was determined as IgG1. Initial analysis of the clone G117-AA4 confirmed its reactivity with the HEV-3 Ctr antigen in ELISA and Western blot analyses (data not shown). In addition to the *E. coli*–expressed HEV-3 Ctr protein, the antibody also detected the corresponding yeast-expressed Ctr protein derivative in both test formats. In addition, the mAb reacted with the O2C antigens of HEV-3 and HEV-1 in the *recom*Line assay (Figure S1). In parallel, human sera were used as negative and positive controls in this line assay.

### Reactivity of the mAb G117-AA4 with HEV-3, ratHEV and batHEV capsid protein derivatives

**Fig. 2:**
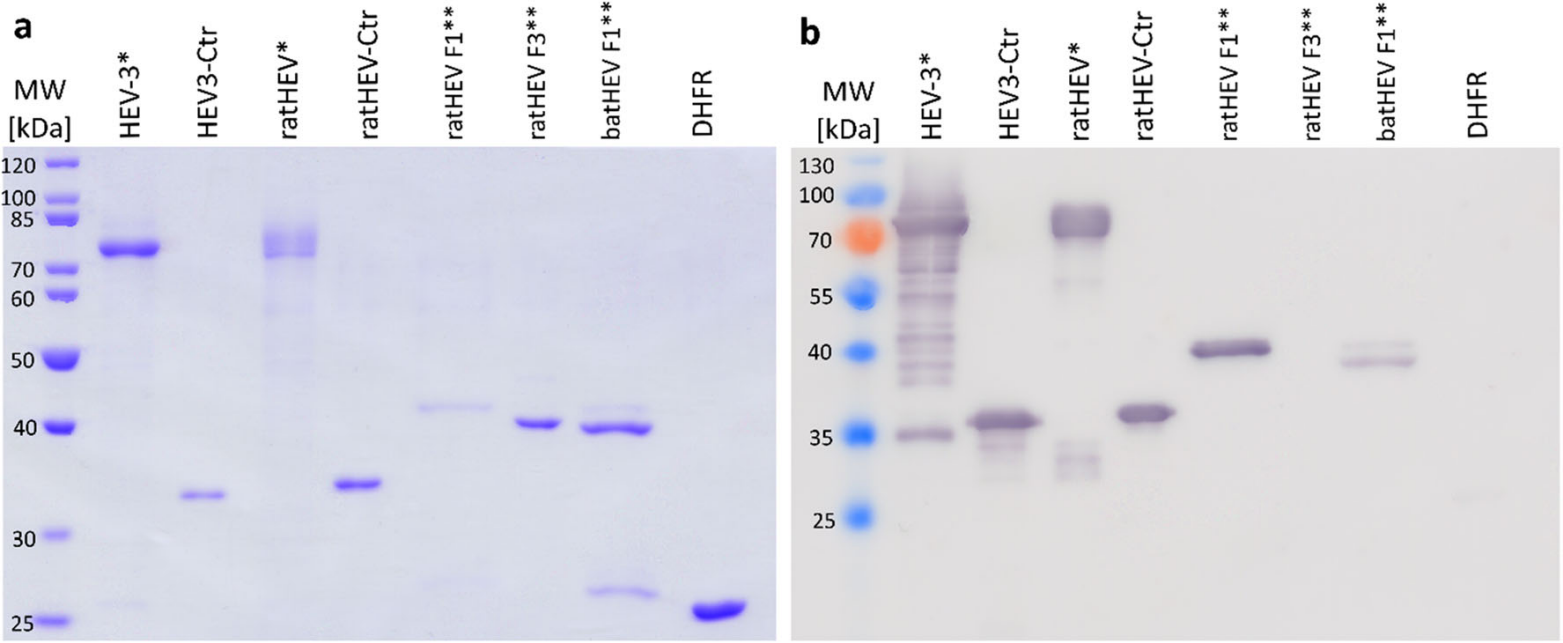
Analysis of recombinant HEV-3, ratHEV, and batHEV capsid protein derivatives by SDS-PAGE (**a**) and their reactivity with G117-AA4 in Western blot (**b**). *HEV* hepatitis E virus, *Ctr* carboxy-terminal truncated capsid protein, *F1* amino-terminal fragment of Ctr, *F3* carboxy-terminal fragment of Ctr, *DHFR* dihydrofolate reductase. * yeast-expressed full-length proteins (else: *E. coli* expressed). ** DHFR-fusion proteins

Clone G117-AA4 showed a strong cross-reactivity to the recombinant *E. coli*–expressed ratHEV and HEV-3 capsid protein derivatives in ELISA (Figure S2). The strong cross-reactivity was confirmed by Western blot analyses (Fig. 2), resulting in similar band intensity of ratHEV-Ctr and HEV-3-Ctr. Besides the detection of these two *E. coli*–expressed recombinant capsid protein derivatives, mAb G117-AA4 successfully detected multiple bands in full-length yeast-expressed ratHEV and HEV-3 capsid proteins. ratHEV and batHEV, for which *E. coli*–expressed recombinant DHFR fusions of Ctr fragments exist (F1 and F3, see Fig. 1c/d), exhibited detection signals exclusively with the F1 part of the protein corresponding to the amino acid residues 316 to 449. The DHFR-F3 constructs of ratHEV did not show any reactivity (Fig. 2). These results were also confirmed by ELISA (data not shown).

### Cross-reactivity of G117-AA4 with cell-free produced capsid protein derivatives

**Fig. 3:**
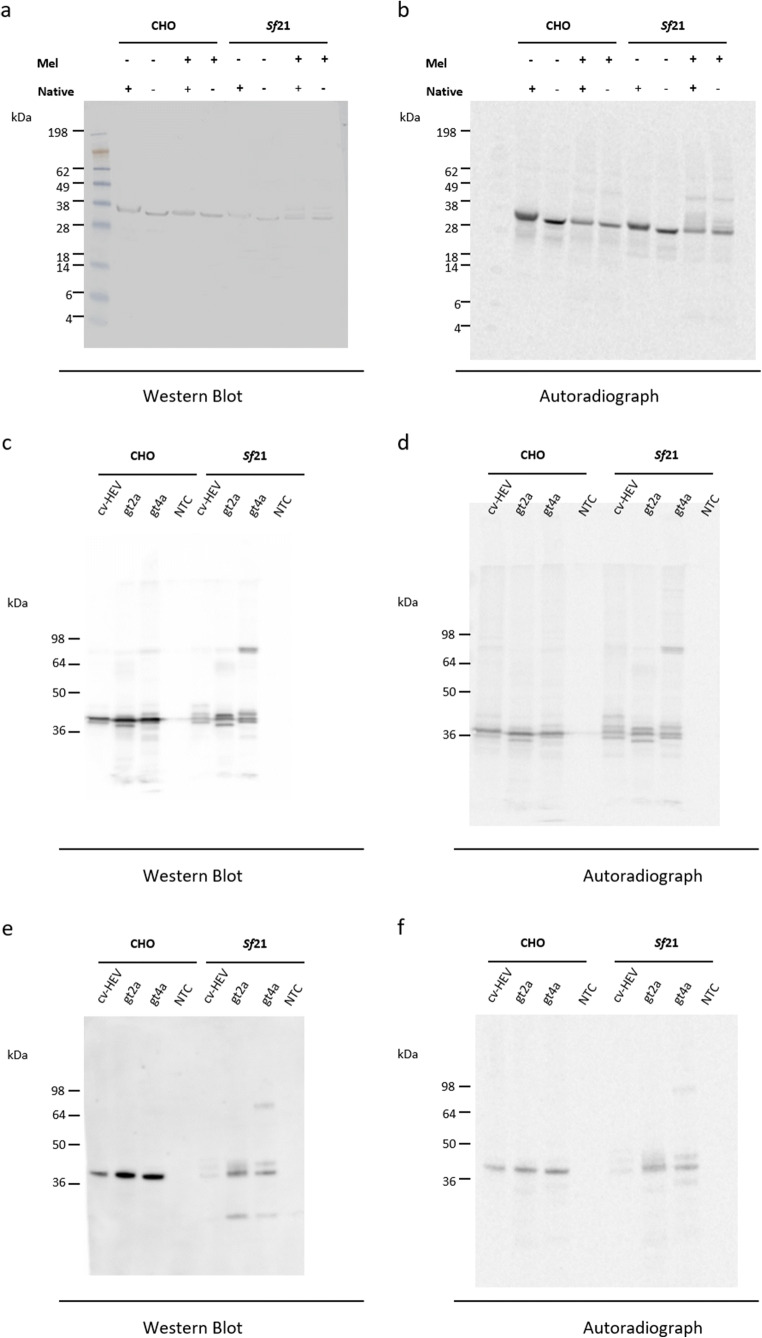
Cell-free synthesis of capsid protein derivatives of common vole HEV (cvHEV), HEV-2 (gt2a) and HEV-4 (gt4a), and detection by Western blot analysis. (**a**) Binding of G117-AA4 antibody to cell-free synthesized cvHEV ORF2-encoded proteins. (**b**) Autoradiography of ^14^C-labelled cvHEV protein visualized on the blot. Constructs with (Mel +) and without (Mel -) the Mel-signal peptide. Proteins were synthesized in CHO and *Sf*21 lysates. Proteins were blotted in a native (+) and precipitated (-) form. (**c**) Binding of G117-AA4 antibody to cell-free synthesized cvHEV, HEV-2 (gt2a) and HEV-4 (gt4a). (**d**) Autoradiography of ^14^C-labelled proteins, initially detected by the monoclonal G117-AA4 antibody, visualized on the blot. (**e**) Western Blot reactivity of polyclonal rabbit serum with cell-free synthesized cvHEV, HEV-2 (gt2a) and HEV-4 (gt4a). (**f**) Autoradiography of ^14^C-labelled proteins, initially detected by the polyclonal serum visualized on the blot. NTC, no template control

The cvHEV C-terminal region of capsid protein was synthesised in CHO as well as *Sf*21 lysates and binding of antibody G117-AA4 was analysed. To characterise the antibody binding, native as well as precipitated protein fractions were analysed. Figure 3a shows the binding of the G117-AA4 antibody to the cvHEV C-terminal region of capsid protein expressed in both lysates, CHO as well as *Sf*21. Further, both native and precipitated proteins could be detected. cvHEV protein harbouring a Mel-signal sequence synthesised in a *Sf*21 lysate showed additional bands that suggested the glycosylation of the protein. To match the blotted protein bands to synthesised radioactively labelled protein, the blot was visualised via autoradiography. Protein bands in the autoradiograph correspond to the blotted proteins (Fig. 3b). The autoradiograph further indicates the glycosylation of cvHEV protein in a *Sf*21 cell-free expression system when using a Mel-signal sequence. In a next step, coding sequences for HEV-2a and HEV-44a capsid protein fragments with a Mel-signal peptide were synthesized in CHO and *Sf*21 lysates in comparison to the cvHEV capsid protein fragment. Figure 3c shows the specific binding of the G117-AA4 antibody to all three HEV protein products synthesized in both CHO and *Sf*21 lysate. Again, the blotted protein bands detected by the antibody correlated to the ^14^C-labelled protein bands in the autoradiograph (Fig. 3d). The control experiment with the polyclonal anti-capsid protein rabbit serum showed its clear binding to the cell-free synthesized proteins (Fig. 3e). ^14^C-labelled proteins were detected by autoradiography to qualitatively confirm the proteins (Fig. 3f).

### Reactivity of G117-AA4 with cell culture–derived HEV-3

**Fig. 4:**
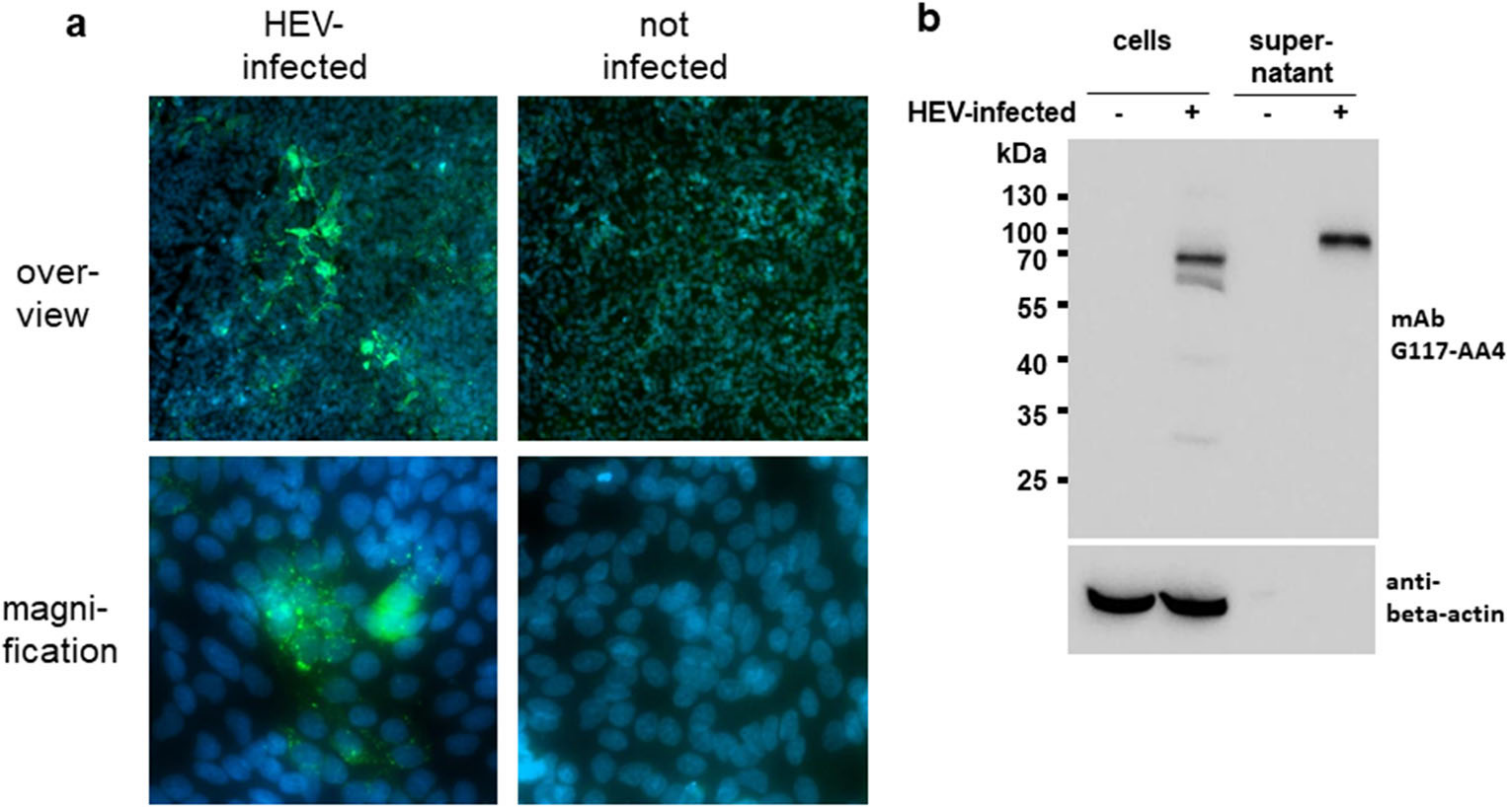
Analysis of A549 cells persistently infected with the HEV-3 strain 47832c and non-infected A549 cells using the antibody G117-AA4. **(a)** Immunofluorescence analysis of infected cells (left) and non-infected cells (right). The lower row shows cells at a higher magnification. Green staining, HEV antigen (antibody G117-AA4); blue staining, cell nuclei (DAPI). **(b)** Western blot analysis of infected and non-infected cells (left) as well as supernatant derived from them (right). The HEV-specific antibody G117-AA4 (upper row) and the anti-β-actin-specific antibody (lower row) were used. The position of molecular weight markers is indicated

By immunofluorescence analysis of persistently HEV-3-infected A549 cell cultures, the antibody G117-AA4 generated several foci of green fluorescent cells with intracytoplasmic staining (Fig. 4a). At a higher magnification, green-stained granules were evident, which were mainly localised in the cytoplasm of the cells (cell nuclei are stained blue). No green fluorescence was observed by testing non-infected A549 cells using this antibody. Analysis of the persistently HEV-3-infected cells by Western blotting using the antibody G117-AA4 resulted in a prominent band with an apparent molecular weight of approximately 70 kDa, along with several weak bands of lower molecular weight (Fig. 4b). In the supernatant of the HEV-infected cells, a single band of approximately 80 kDa was evident. No bands were detected using this antibody in non-infected cells or supernatants derived from them. As expected, the cellular marker beta-actin was detected by another specific antibody in infected and non-infected cells with the same intensity, but not in the supernatants derived from them.

### Cross-reactivity of mAb G117-AA4 to hare HEV-3 and camel HEV-7 capsid proteins

**Fig. 5:**
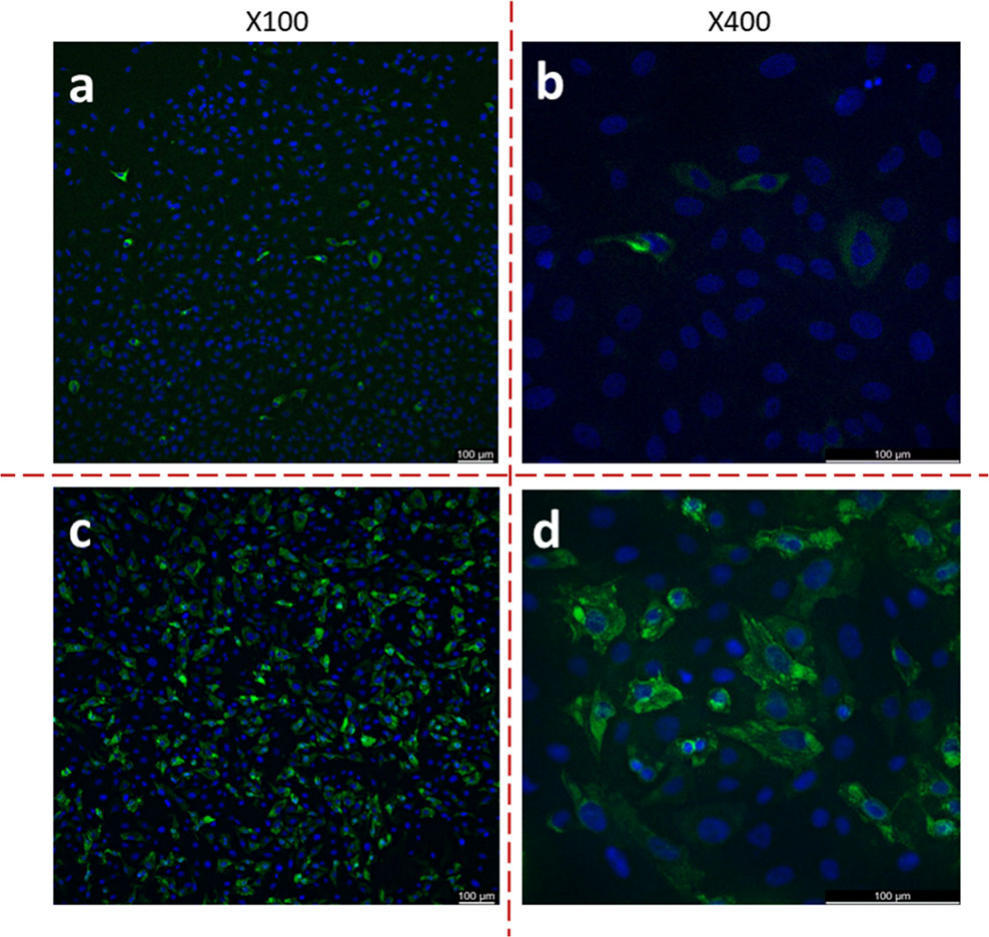
Immunofluorescence assay–based detection of the reactivity of antibody G117-AA4 with Vero B4 cells transfected with pCG1-derived expression plasmids encoding hare HEV-3 **(a, b)** and camel HEV-7 **(c, d)** capsid proteins. The monoclonal antibody was diluted 1:100. The anti-mouse IgG secondary antibody was Alexa 488-labelled. Slides were counterstained by DAPI. Magnifications are given above the pictures

Immunofluorescence analysis of Vero B4 cells transfected by eukaryotic expression plasmids confirmed the reactivity of mAb G117-AA4 with capsid proteins of hare HEV-3 and camel HEV-7 (Fig. 5a, c). The analysis of the immunofluorescence slides at higher magnification confirmed the specificity of the reaction by the typical cytoplasmic staining (Fig. 5b, d).

### Pepscan-based epitope mapping

**Fig. 6:**
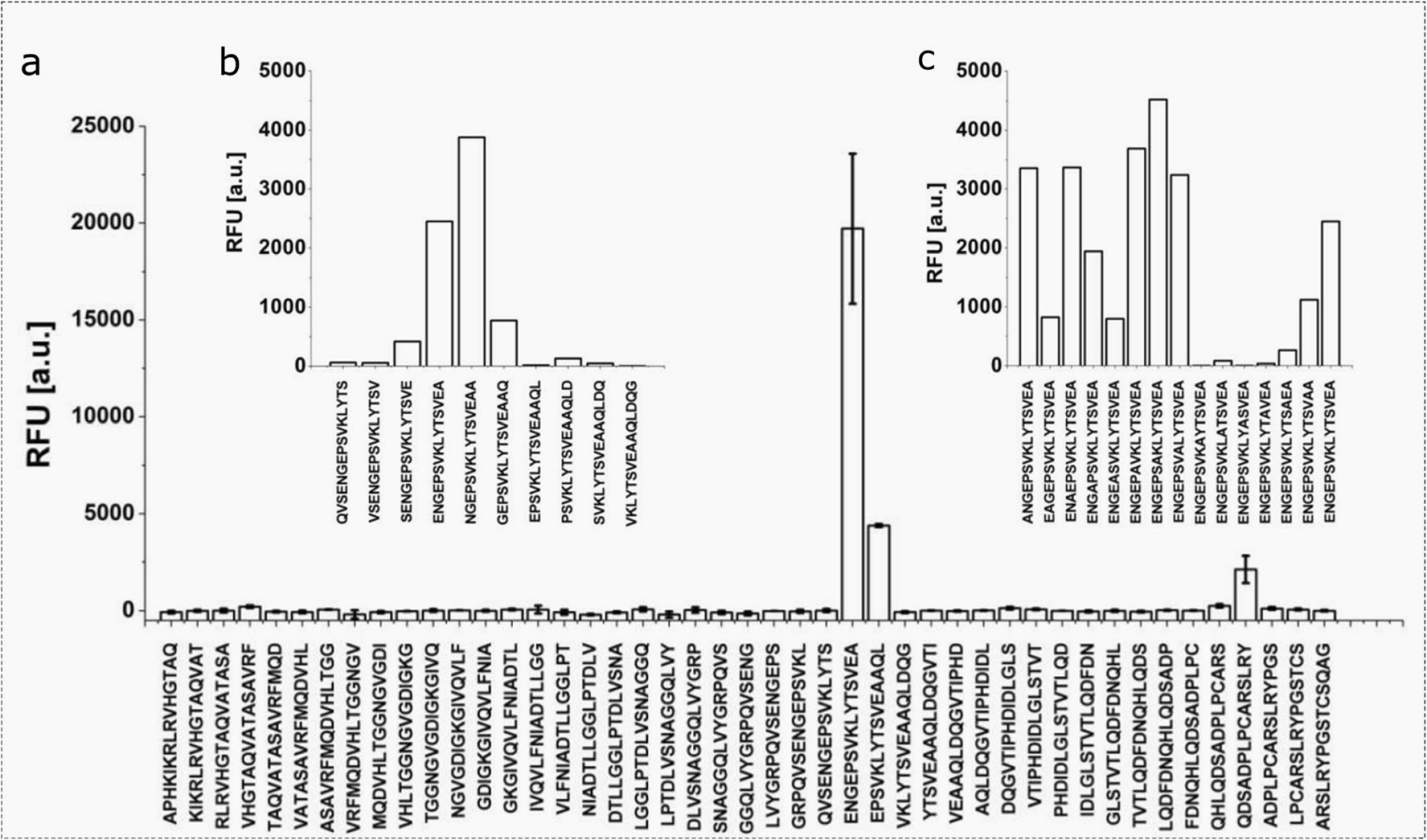
Epitope mapping of ratHEV-F1 in 15 aa peptides with an offset of 3 aa (**a**). Two regions exhibited relative fluorescence higher than 1000 RFU (1. ENGE…AAQL and 2. QHLQ…YPGS). The weaker signal (peptide QHLQ…YPGS) could be traced back to non-specific binding of the secondary antibody. Epitope mapping with an offset of 1 aa (**b**) and subsequent replacement of single amino acid residues by alanine (**c**) revealed the sequence LYTSV to be essential for proper binding of mAb G117-AA4

Further refinement of the epitope region was achieved by scanning the full-length ratHEV-F1 sequence in 15-amino acid peptides with an offset of 3 amino acids for antibody binding. Two regions exhibited fluorescence signals higher than 1000 relative fluorescence units (RFU; Fig. 6a). The weaker signal, referring to the sequence QDSADPLPCARSLRY, could be traced back to non-specific interaction of the secondary antibody with the peptide. The sequence exhibiting a stronger fluorescence was further scanned with an amino acid offset of 1, resulting in the sequence NGEPSVKLYTSVEAA (Fig. 6b, ratHEV capsid protein: 394-408 aa) giving the strongest signal. Subsequent replacement of the amino acid residues within this sequence by alanine revealed the positions LYTSV to be essential for proper binding of mAb G117-AA4 (Figure 6c).

### Amino acid sequence comparison and epitope localisation

**Fig. 7:**
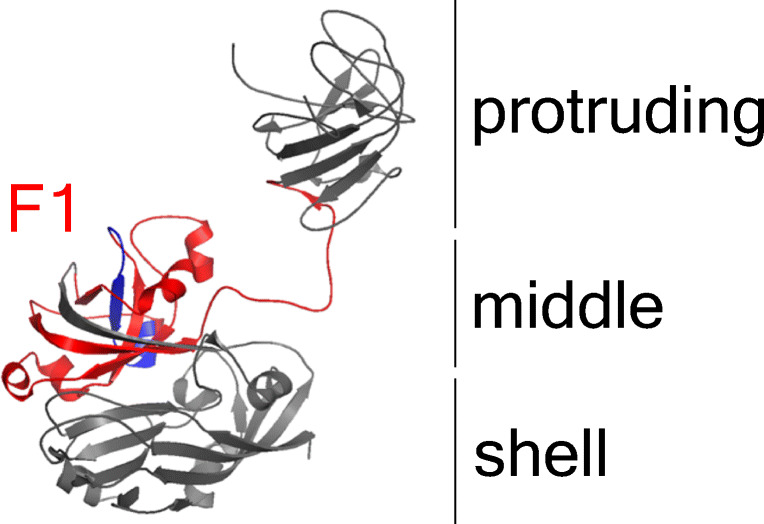
Epitope localization in HEV-3 capsid protein monomer. Model was calculated with PyMOL based on the crystal structure 2ZTN (Yamashita et al. 2009). Capsid protein domains are named according to Yamashita et al. (2009). Rat HEV-F1 homologous region (red) and NGEPSVKLYTSVEAA-sequence (assumed epitope, dark blue) are highlighted

The conservation of the suspected epitope region was examined via multiple amino acid sequence alignment (MSA) of different HEV species, genotypes and strains (Table 2). Divergence from the sequence LYTSV, appearing to be essential for mAb G117-AA4 binding in the epitope mapping experiment, was only observed for batHEV Ctr_BS7_GE_2009 (GenBank: JQ001749.1) with one amino acid substitution and for avian HEV (GenBank: AY535004). Structural modelling of the HEV-3 capsid protein indicates localisation of the epitope at an outer pocket of its middle domain (Fig. 7).

## Discussion

In the present study, we generated and characterised a HEV-3-capsid protein specific mAb with strong cross-reactivity to capsid proteins of HEV-1, HEV-2, hare HEV-3, HEV-4, HEV-7, ratHEV, cvHEV and, to a lower extent, also to batHEV capsid protein. Cross-reacting HEV-specific antibodies have also been described previously (He et al. 2007; Kobayashi et al. 2016; Riddell et al. 2000; Schofield et al. 2000, 2003; Takahashi et al. 2008a; Zhang et al. 2005). Despite great genetic diversity, HEV-1–HEV-7 and rabbit HEV, including the human-pathogenic genotypes (HEV-1–HEV-4), are thought to represent a single serotype (Acharya and Panda 2006; Behloul et al. 2015; Boyer et al. 2012; Lee et al. 2016; Li et al. 2015, 2016; Perez-Gracia et al. 2015; Schlauder and Mushahwar 2001; Smith et al. 2014; Schofield et al. 2003; Wang et al. 2013). Nevertheless, modelling data suggest a slightly different surface exposition of the epitopes, probably accounting for some of the differences in specificity, sensitivity and inter-assay detection observed with diagnostic antigens derived from different genotypes (Behloul et al. 2015; Drobeniuc et al. 2010).

MAb G117-AA4-related signals of affinity for capsid proteins from HEV-1, HEV-2, HEV-3, HEV-4, ratHEV, batHEV and cvHEV in Western and line blot analysis showed strong cross-reactivity. In addition to the strong cross-reactivity, mAb G117-AA4 reacted with *E. coli*–expressed and yeast-expressed HEV proteins and native viral antigen, indicating that the binding occurs independently of posttranslational modification. Immunofluorescence staining of persistently HEV-3-infected cell culture and cells transfected by hare HEV-3 and camel HEV-7 capsid protein expression plasmids revealed mainly cytoplasmic viral proteins (Fig. 4, Fig. 5). The successful staining of native antigen by immunofluorescence indicates at least a partial surface exposition and consequently accessibility of the epitope. Furthermore, the reactivity of the antibody with recombinant and viral antigen in the Western blot analysis suggested a linear epitope as a binding site.

Using a truncated ratHEV capsid protein derivative, the epitope region could be mapped to amino acid residues 316-449 by Western blot detection. Additionally, mAb G117-AA4 recognised the analogous batHEV F1 construct in ELISA and Western blot analysis, underlining the cross-reactivity and epitope conservation. Further epitope mapping by Pepscan analysis of ratHEV identified amino acids 394-408 as the binding site, and the alanine scan revealed the sequence LYTSV as an essential binding site. This highly conserved region is located within the scaffolding middle region of the capsid monomer, and protein modelling of the capsid structure shows that this epitope is partially exposed to the surface (Wang et al. 2015). Nevertheless, synergistic effects of antibody binding and epitope accessibility have been reported in competitive studies (Zhang et al. 2005) possibly altering surface exposure of epitopes. Since mAb G117-AA4 successfully detected viral protein by non-denaturating immunofluorescence analysis of persistently infected cell culture, the epitope appears to be accessible at some stages of virion assembly. Also, the release of the virion as a lipid-associated particle has been proposed, modulating epitope accessibility, antibody-binding and neutralisation of virus infectivity (Feng and Lemon 2014; Takahashi et al. 2008a, b).

Immunofluorescence analysis using HEV-3 strain 47832c-infected A549 cells and the novel mAb indicated mainly intracytoplasmic staining of cells and granulae typical for HEV infection as shown in previous studies (Johne et al. 2014b; Takahashi et al. 2008b). By Western blot analysis, a prominent band of 70 kDa was detected in the cells and a band of 80 kDa in the supernatant, which correspond to the described 74 kDa non-glycosylated capsid protein and the 82 to 88 kDa glycosylated capsid proteins, respectively (Jameel et al. 1996). In line, glycosylation was found during cell-free synthesis of Mel-signal sequence-harbouring cvHEV, HEV-2a and HEV-4a capsid proteins in *Sf*21 lysate as seen in Fig. 3. Our finding is in line with recently published results, showing that the non-glycosylated capsid protein of HEV is mainly found within cells, whereas glycosylated forms of this protein are actively secreted into the culture supernatant (Yin et al. 2018). These results underline the suitability of the mAb for HEV cell culture studies.

In conclusion, the novel mAb represents a useful tool for detecting HEV antigen in infected cells for pathogenicity and transmission studies and might be of high value for future diagnostic applications. The observed cross-reactivity may allow its use with more distantly related members of the genus *Orthohepevirus*. Further epitope mapping, cross-reactivity studies and structural studies on different HEV capsid proteins are needed to understand the structural basis of the antigenicity of the epitope region.

## Supplementary Information

ESM 1 (DOCX 308 kb).

## Data Availability

The datasets generated during the current study are included in this published article and its supplementary information file and are available from the corresponding author on reasonable request.
