# Supplementary material for: A broadly cross-reactive monoclonal antibody against hepatitis E virus capsid antigen
Source: Appl Microbiol Biotechnol. 2021 Jun 15;105(12):4957–73. doi: 10.1007/s00253-021-11342-7 (PMC8236046; doi:10.1007/s00253-021-11342-7)
Supplement: Supplementary file 1 — (DOCX 308 kb). [file 253_2021_11342_MOESM1_ESM.docx]

# Applied Microbiology and Biotechnology

# A broadly cross-reactive monoclonal antibody against hepatitis E virus capsid antigen

Barbara Kubickova^1,2#^, Jörg A. Schenk^3,4^, Franziska Ramm^5,6^, Kornelija Markuškienė^1,7^, Jochen Reetz^8^, Paul Dremsek^1,9#^, Paulius Lukas Tamosiunas^7^, Laima Cepulyte^7^, Hoai Anh Trinh^5^, Johannes Scholz^8^, Henry Memczak^10^, Marc Hovestädt^10,11#^, René Ryll^1^, Rasa Petraityte-Burneikiene^7^, Victor M. Corman^12,13^, Anika Andersson^4,5^, Dietmar Becher^15^, Martin H. Groschup^1,14^, Stefan Kubick^5,6,16^, Frank Sellrie^3,4^, Reimar Johne^8^, Rainer G. Ulrich^1,14,*^

^1^Institute of Novel and Emerging Infectious Diseases, Friedrich-Loeffler-Institut, Federal Research Institute for Animal Health, , 17493 Greifswald-Insel Riems, Germany

^2^RECETOX, Faculty of Science, Masaryk University, 62500 Brno, Czech Republic

^3^Hybrotec GmbH, 14476 Potsdam, Germany

^4^UP Transfer GmbH an der Universität Potsdam, 14469 Potsdam, Germany

^5^Branch Bioanalytics and Bioprocesses (IZI-BB), Fraunhofer Institute for Cell Therapy and Immunology (IZI), 14476 Potsdam, Germany

^6^Institute of Chemistry and Biochemistry, Freie Universität Berlin, 14195 Berlin, Germany

^7^Institute of Biotechnology, Life Sciences Centre, Vilnius University, 02241 Vilnius, Lithuania

^8^German Federal Institute for Risk Assessment, 10589 Berlin, Germany

^9^Zentrum für Pathobiochemie und Genetik, Medizinische Universität Wien, 1090 Wien, Austria

^10^qpa bioanalytics GmbH, 10585 Berlin, Germany

^11^Surflay Nanotec, 12489 Berlin, Germany

^12^Institute of Virology, Charité – Universitätsmedizin Berlin, 10117 Berlin, Germany

^13^ Site Berlin, German Center for Infection Research (DZIF), 10117 Berlin, Germany

^14^ Partner site Hamburg-Lübeck-Borstel-Riems, German Center for Infection Research (DZIF), 17493 Greifswald-Insel Riems, Germany

^15^Micromun GmbH, 17489 Greifswald, Germany

^16^aculty of Health Sciences, Joint Faculty of the Brandenburg University of Technology Cottbus – Senftenberg, the Brandenburg Medical School Theodor Fontane and the University of Potsdam, Germany

* Corresponding author. Tel. +49 38351 7 1159

E-mail address: rainer.ulrich@fli.de

# Supplementary material


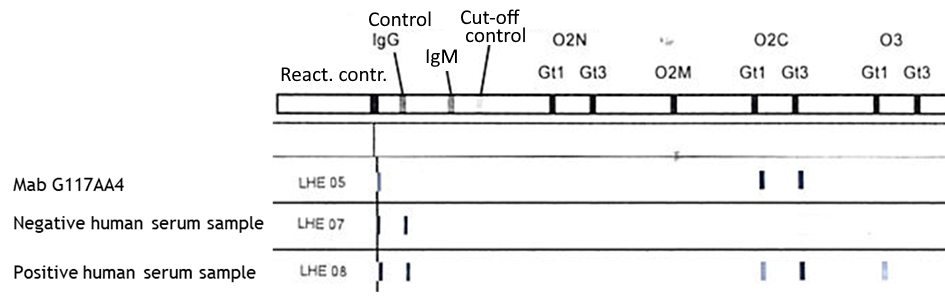


**Fig. S1.** Analysis of mAb G117-AA4 in commercial *recom*Line assay (Mikrogen). For control a positive human serum (strip LHE 08) and a negative human serum (strip LHE 07) were used. The secondary antibodies were horseradish peroxidase-labeled anti-mouse IgG for the mAb and anti-human-IgG for the human sera.


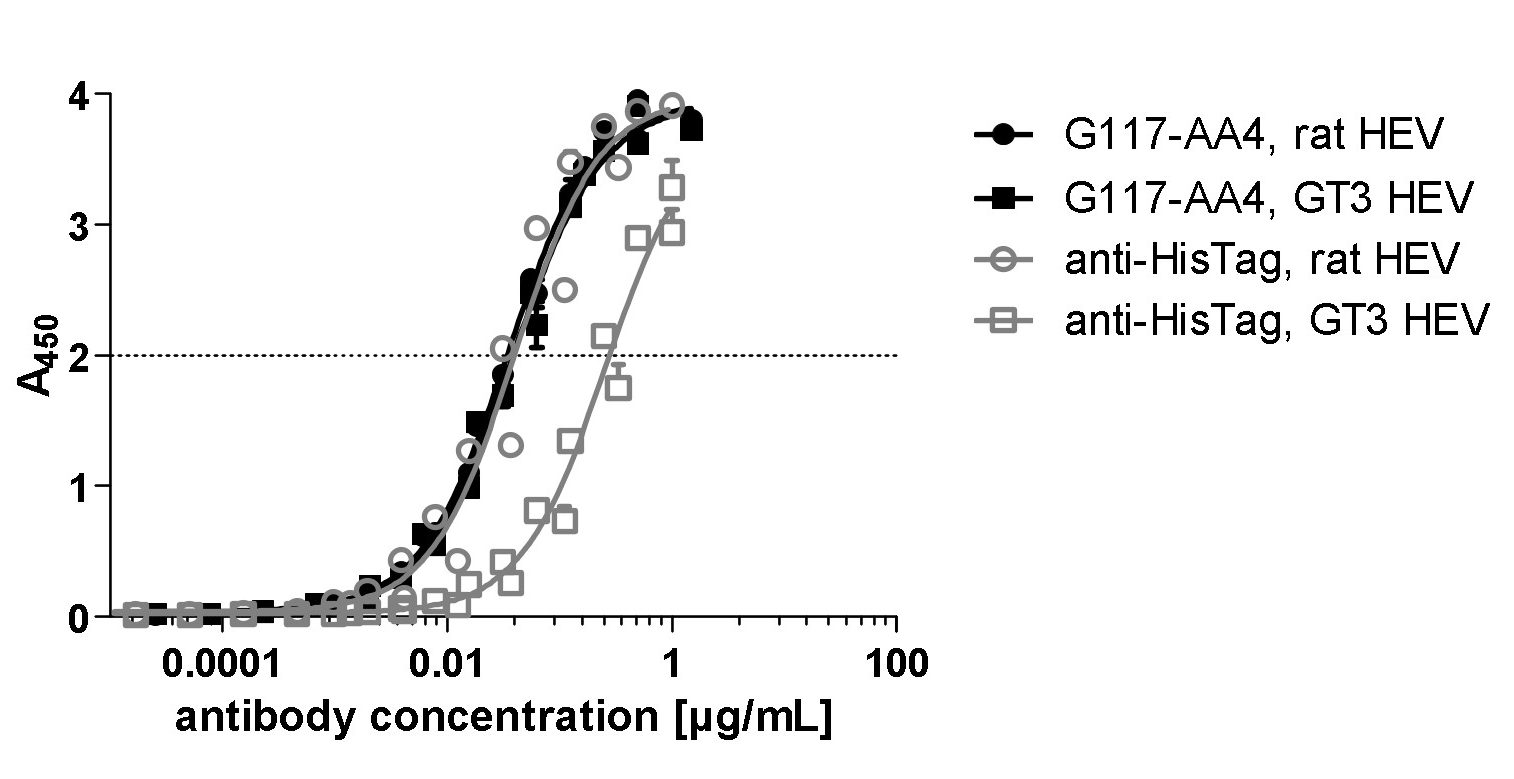


**Fig. S2.** ELISA titration of G117-AA4 (filled marks) in comparison to a commercially available antibody against the aminoterminal decahistidine-tag of HEV-3 and ratHEV Ctr antigens (empty marks; Novagen, Germany). Half-maximal absorption values (EC_50_), indicated by the dotted line, are 0.0340 µg/ml (G117-AA4, rat HEV), 0.0383 µg/ml (G117-AA4, HEV-3), 0.0401 µg/ml (anti-His tag, rat HEV) and 0.2572 µg/ml (anti-His tag, HEV-3). Mean values of two replicates are shown.
